# Retinal vascular reactivity is associated with white matter hyperintensities and dysfunctional cerebrovascular reactivity in cerebral small vessel disease

**DOI:** 10.1177/0271678X251366079

**Published:** 2025-08-06

**Authors:** Gordon W Blair, Ian J C MacCormick, Sarah McGrory, Tom MacGillivray, Iona Hamilton, Yulu Shi, Francesca Chappell, Michael J Thrippleton, Michael S Stringer, Fergus Doubal, Joanna M Wardlaw

**Affiliations:** 1 Centre for Clinical Brain Sciences, University of Edinburgh, UK; 2 Department of Stroke and Medicine for the Elderly, Royal Infirmary Edinburgh, UK; 3 Robert O Curle Ophthalmology Suite, Institute for Regeneration and Repair, University of Edinburgh, UK; 4 Row Fogo Centre for Research into Ageing and the Brain, University of Edinburgh, UK; 5 Institute for Adaptive and Neural Computation, University of Edinburgh, UK; 6 Department of Psychology, School of Philosophy, Psychology and Language Sciences, University of Edinburgh, UK; 7 UK Dementia Research Institute Centre, University of Edinburgh, UK; 8 Edinburgh Imaging, University of Edinburgh, UK

**Keywords:** Cerebral small vessel disease, cerebrovascular reactivity, magnetic resonance imaging, retinal imaging, retinal vessel reactivity

## Abstract

Cerebral small vessel disease (cSVD) is characterised by white matter hyperintensities (WMH) and contributes to stroke and dementia. Cerebrovascular reactivity (CVR) declines with worsening cSVD but estimates of CVR from brain scans give limited information about the coordination of individual arterioles or venules. Retinovascular reactivity can provide separate data about arterioles and venules, but it is not known if retinovascular reactivity correlates with CVR in people with cSVD. We aimed to assess retinal vascular reactivity in people with cSVD and explore associations with WMH and CVR in a cross-sectional study. Participants had retinal photographs and magnetic resonance brain imaging (MRI) before and during inhalation of 6% CO_2_ in air. We recruited 60 participants. 48 provided analysable retinal vessel reactivity data. Retinal arteriole/venule ratio was inversely related to WMH severity (adjusted R2 = 0.47 β–5.2 95%CI–7.9 to −2.5) and directly related to CVR (adjusted R2 = 0.21 β0.15 95%CI 0.04 to 0.25). In general, participants whose arteriole/venule ratio increased with CO_2_ had milder WMH and higher (better) CVR, while participants whose arteriole/venule ratio decreased had more severe WMH and lower (worse) CVR. Retinovascular reactivity to CO_2_ inhalation in people with cSVD suggests loss of normal arteriovenous coordination and inefficient perfusion of the capillary bed.

## Introduction

Cerebral small vessel disease (cSVD) has several radiological features, including white matter hyperintensities (WMH), lacunes, microhaemorrhages, and perivascular spaces,^1,2^ which may result in symptoms. cSVD contributes to substantial proportions of stroke and cognitive impairment, and cerebrovascular dysfunction appears to be an early tractable microvascular abnormality.^1^ Therefore, measuring microvascular dysfunction within the central nervous system could help us understand pathophysiological mechanisms^1^ and help identify and measure the effects of novel interventions.^3^

Cerebrovascular reactivity (CVR) to inhaled carbon dioxide (CO_2_) reflects the chemo-regulatory function of microvessels to dilate,^4^ is related to the severity of cSVD features,^5–7^ may predispose to future development of brain lesions,^8^ and appears to respond to interventions targeting vascular endothelial function.^9,10^ CVR can be assessed using the blood oxygen level dependent magnetic resonance imaging (BOLD MRI) response to 6% inhaled CO_2_.^11^ However, neither BOLD MRI nor conventional structural MRI are able to resolve individual microvessels at the arteriole, capillary or venule level – the epicentre of cSVD pathogenesis. Transcranial doppler ultrasound can estimate CVR as change in velocity in one large artery, usually the middle cerebral artery, but is operator-dependent, limited by acoustic window, and gives an average blood flow response for the whole arterial territory.^12^

Blood vessels of the retina are visible on ophthalmoscopy, and share embryology, anatomy and physiology with the brain vasculature. Like the cerebrovasculature, retinal microvessels serve neural tissue that is highly metabolically active and reliant on a constant supply of oxygen and glucose. This explains broad similarities in neurovascular unit structure, blood-barrier function and microvascular geometry between retina and brain,^13^ as well as similar capacities to regulate local blood flow through chemo-regulation (e.g., reactivity to CO_2_), neurovascular coupling, and autoregulation.^4,14–17^

Retinal blood flow response to CO_2_ has been estimated at ∼3% per mmHg PaCO_2_ in non-human primates^18^ (reviewed in ^19^) and ∼2.5% in humans.^20^ This is broadly similar to the lower range of estimates of human cerebral blood flow response to CO_2_ (∼3 to 6% per mmHg PaCO_2_) (reviewed in^4^). There are few studies directly comparing retinal and cerebrovascular reactivity to CO_2_ in the same participants.^15,21^ These did not show associations between flow velocity response to CO_2_ in the ophthalmic or central retinal arteries and middle cerebral artery, possibly due to different relative contributions to blood flow from velocity and vessel widening between these vessel segments which have very different diameters (∼150 µm and ∼3 mm for central retinal and middle cerebral arteries, respectively^22,23^)

Retinopathy and *static* retinal vessel calibres (narrower arterioles, wider venules) are associated with lacunar stroke,^24,25^ WMH,^26,27^ and vascular dementia^28,29^ in longitudinal studies and meta-analyses. However, although *dynamic* retinal vessel reactivity to CO_2_ or light has been studied in other populations^19,30–32^ it is not clear whether retinovascular reactivity might reflect neuroradiological signs of cSVD^30,33,34^ such as WMH; or CVR, which declines as WMH increase.^5^

One small case-control study (n = 12 with WMH, n = 14 controls) has suggested a possible association between impaired retinovascular reactive dilation to flickering light, WMH, and impaired middle-cerebral artery flow velocity measured by transcranial doppler ultrasound, in response to alternating hyperventilation and breath-holding.^34^ They found a direct association between reactivity of retinal venules and middle cerebral artery (r = 0.45, p = 0.045), and that participants with white matter hyperintensities had significantly lower retinal flicker-induced dilation comparted to healthy controls (p < 0.01 for both retinal arterioles and venules).^34^

If retinal and cerebrovascular reactivity are affected analogously by cSVD, and if impairments in reactivity have similar associations with WMH, then this would suggest dynamic retinal vessel reactivity could help provide insights into, and possibly monitor, cSVD pathophysiology.

We hypothesised that in people with cSVD the response of retinal vessel diameters to CO_2_ is associated with WMH severity and CVR. We aimed to investigate the feasibility of colour fundus photography during CO_2_ inhalation in people with cSVD and explore associations between changes in retinal vessel widths and neuroradiological and systemic variables.

## Methods

### Study design

This is a cross-sectional study reporting retinal data from a cohort described previously.^5^ All participants provided written informed consent before recruitment. The study was approved by the UK Health Research Authority National Research Ethics Service Committee East Midlands, Nottingham 1 (ref. 14/EM/1126). We followed STROBE guidelines adapted to pilot/feasibility studies.^35^ The study adhered to the principles of the Declaration of Helsinki.

### Setting

Regional stroke service provided by the National Health Service, Lothian, UK.

### Participants

Patients presenting with symptomatic nondisabling ischemic stroke (modified Rankin Scale score ≤3)^36^ between October 2014 and April 2016 were eligible. Participants of recent previous stroke studies were also invited to participate.^37^ All participants had a clinical diagnosis of stroke and brain imaging ruling out alternative causes for their symptoms. Stroke syndrome was classified independently by two stroke physicians (FND and GWB),^38^ and brain lesion type (independently by JMW and YS). Discrepancies were resolved by discussion. If radiological lesion type conflicted with clinical classification, lesion type informed the final classification unless no radiological lesion was visible.^39^ Exclusion criteria were: pregnancy, contraindications to MRI, moderate to severe chronic respiratory disease or symptomatic cardiac failure, uncontrolled hypertension or atrial fibrillation with fast ventricular response, personal history or first-degree relative with subarachnoid haemorrhage or intracranial aneurysm, and no view of the retina in both eyes.

### Variables and sources of data

Study assessments were done >1 month after the participant’s stroke to limit the effect of acute disease and initiation of new stroke secondary prevention drugs on cerebrovascular function. Participants were asked not to consume caffeine on the day of assessment. Each participant had retinal photography and brain MRI during the same research session. Details of clinical assessments, MRI sequences, and MRI analyses are in Blair et al., 2020.^5^

**Figure 1.**
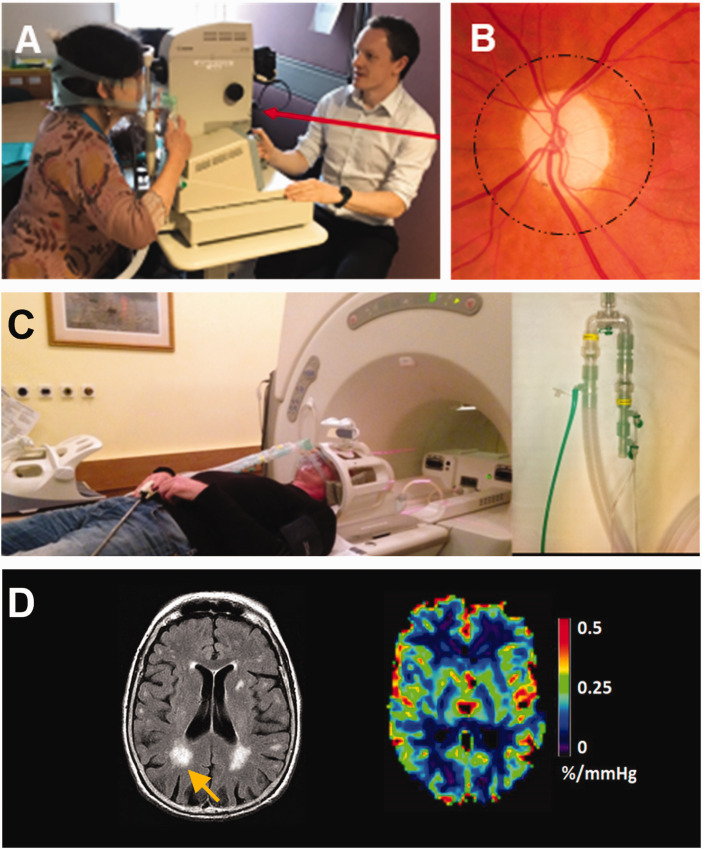
a. Retinal photography performed with a mask and breathing circuit. b. This captures retinal vessels around the optic disc, which are used to calculate the central retinal artery, and central retinal vein equivalents (CRAE, CRVE) and artery-vein ratio (AVR). c. MRI with mask and breathing circuit. D. MRI images show white matter hyperintensities (WMH) among other imaging features of cerebral small vessel disease. BOLD MRI with CO_2_ challenge allows cerebrovascular reactivity (CVR) to be estimated.

Forty-five-degree retinal images centred on the optic disc were taken of one eye per participant (the left eye in 43/44 participants) using a table-mounted non-mydriatic fundus camera (CR-DGi, Canon USA) and stored at a resolution of 3504 × 2336 pixels in bitmap format (Figure 1). The pupil was not dilated before imaging. Retinal images were taken with a tight-fitting face mask in place, first while breathing air and then at the end of a 90 second period of medical air (21% O_2_ + 73% N_2_) + 6% CO_2_ gas mixture through a unidirectional breathing circuit (Intersurgical, Wokingham, UK) (Figure 1). Fundus photography was achieved by having the participant look slightly leftwards and angling the camera nasally for left eye photographs (vice versa for the right eye). The order of gasses was not randomized, and investigators were not masked to gas status. Participants tried the facemask before retinal imaging commenced, were told to expect a change in smell and faster deeper breathing. Participants were monitored by a physician during gas inhalation.

A subset of participants (n = 8) had baseline images taken twice (within minutes) to assess intra-individual variation in vessel widths breathing air. Retinal images were also repeated immediately when initial image quality was judged to be poor by the photographer (n = 6).

Retinal imaging was immediately followed by MRI to assess structural features of cSVD, CVR by BOLD MRI, and phase contrast MRI to assess arterial, venous and CSF blood flow and pulsatility. Briefly, brain MRI was performed with a 1.5 Tesla research scanner (Signa HDxt, General Electric, Milwaukee, WI) in research mode with an 8-channel phased array head coil. 3 D T1-weighted, axial T2-weighted, fluid-attenuated inversion recovery (FLAIR) and gradient echo sequences were acquired. BOLD MRI was done at 4 mm isotropic resolution, acquiring the whole brain every 3 seconds.^40^

CVR was measured with a gas paradigm of 2 min Medical Air, followed by 3 min 94% Medical Air + 6% CO_2_, followed by 2 min Medical Air, 3 min 94% Medical Air + 6% CO_2_, followed by 2 min Medical Air. Gas was administered through a similar circuit used for retinal imaging, after similar instructions, checks and safety procedures. Scanning was started only after the patient was comfortable with stable cardiovascular readings.^5^ Retinal and brain imaging methods are illustrated in Figure 1.

### Image processing and analysis

All image processing and analyses were done masked to participant demographic, clinical, and outcome data, as well as corresponding retinal or MRI data and inhaled gas.^5^ Retinal vessel widths were measured using semi-automated software (VAMPIRE [Vessel Assessment and Measurement Platform for Images of the REtina], Universities of Edinburgh and Dundee, UK).^41^ For each eye, the central retinal artery equivalent (CRAE) and central retinal vein equivalent (CRVE) were calculated within VAMPIRE from the six largest vessels between 0.5 and 1 disc diameter from the edge of the optic disc, using a version of the Parr-Hubbard formula.^42^ The artery-vein ratio (AVR) for each image was calculated as AVR = CRAE/CRVE. Change in retinal metrics with CO_2_ inhalation was calculated as the difference between values measured during CO_2_ inhalation and values measured breathing air. For example, change in AVR (ΔAVR) = AVR_CO2_ – AVR_Air_. CRAE and CRVE are reported in pixels and were not adjusted for eye length or focal power.

cSVD features were graded according to STRIVE criteria (STandards for ReportIng Vascular changes on nEuroimaging)^43^ under the supervision of an expert neuroradiologist (JMW). Two graders independently scored features: WMH using the Fazekas scale, summing periventricular and deep scores to give a total score from 0 to 6; perivascular spaces; lacune location and number; microbleed number; atrophy score; and total cSVD score, as described in Blair et al., 2020.^5^ WMH volume was calculated from co-registered structural images (FLAIR and T1W) using a semiquantitative technique.^5,44^ Intracranial volume was calculated from T2*W images, and WMH volume was log-normalised to intracranial volume. CVR data were processed as described previously^40^ as the % BOLD signal change breathing CO_2_ per unit of end-tidal CO_2_ (EtCO_2_) change adjusted for time delay between BOLD signal change and EtCO_2_.^5,11^ CVR was calculated from the mean signal in three subcortical grey matter and four subcortical white matter regions of interest. These were averaged to give combined deep grey matter and white matter CVR values.^5^

### Potential sources of bias

**Figure 2.**
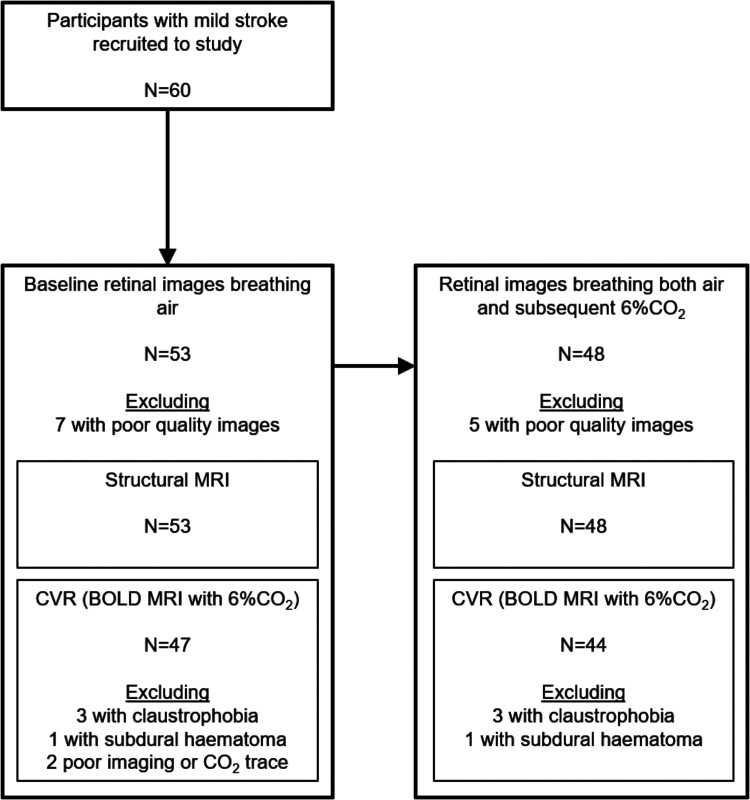
Study participants. All those excluded from retinal measures had poor quality images that could not be analysed. Demographics for the group (n = 53) with baseline retinal images and structural MRI, and group (n = 44) with retinal and brain imaging during CO_2_ challenge, are in Table 1. Reasons for missing MRI data included: claustrophobia, incidental subdural haematoma, poor EtCO2 trace, imaging artefact, poor quality gating.

Patients presenting with mild stroke to our centre and willing to take part in research may not reflect the population of people with mild stroke in general. Not all of the recruited participants were able to complete retinal or brain imaging. Statistics summarising the demographics and image characteristics of the derived sample (Figure 2) are in Table 1 and Supplementary Information to aid comparisons with other samples. Our sample has more men than women, which is seen in several hospital-based studies of SVD.^45^

**Table 1.** Characteristics of 53 people who had acceptable baseline retinal imaging, of whom 47 also had BOLD MRI. Characteristics of 44 people who had both retinal imaging and BOLD MRI with and without CO_2_, corresponding to the bottom right square in Figure 1.

Variable	Retinal imaging breathing air, +/− BOLD MRI CO_2_ challenge (n = 53^a^)	Retinal imaging and BOLD MRI with CO_2_ challenge (n = 44)
Age, years, mean (SD)	67.8 (8.7)	67.1 (8.7)
Male, %	75	75
Diabetes, %	13	11
Hypertension, %	77	77
Hypercholesterolaemia, %	66	61
Ischaemic Heart Disease, %	11	9
Smoking History (%): never	45	43
ex-greater 1 yr	34	34
ex-less 1 yr	4	5
current	17	18
Alcohol Excess, %	19	20
Alcohol, Units per week, median (IQR)	7 (1 to 15)	8 (1 to 18)
Systolic BP, mmHg, mean (SD)	143 (17)	142 (16)
Diastolic BP, mmHg, mean (SD)	80.9 (9.1)	79.6 (8.4)
Lacunar subtype, %	64	64
Days between index stroke and study scan, median (IQR)	107 (54 to 1430)	105 (55 to 1410)
White matter hyperintensity volume, ml, median (IQR)	10.0 (5.7 to 24.1)	9.1 (5.4 to 25.2)
WMH volume, % of intracranial volume, median (IQR)	0.65 (0.42 to 1.55)	0.64 (0.38 to 1.64)
Total Fazekas Score, %: 0	2	0
1	8	7
2	38	41
3	13	11
4	15	18
5	11	9
6	13	14
CRAE, pixels, median (IQR)	30.7 (28.1 to 32.6)	30.7 (28.5 to 32.7)
CRVE, pixels, median (IQR)	40.4 (37.1 to 43.0)	40.2 (37.2 to 42.8)
AVR, pixel ratio: CRAE/CRVE, median (IQR)	0.75 (0.7 to 0.8)	0.75 (0.7 to 0.8)
ΔCRAE, pixels, median (IQR)	n/a	−0.3 (−1.7 to 0.7)
ΔCRVE, pixels, median (IQR)	n/a	−0.7 (−2.9 to 2.4)
ΔAVR, pixel ratio, median (IQR)	n/a	0.01 (−0.04 to 0.05)
Deep Grey Matter CVR Magnitude, %/mmHg, median (IQR), in n = 47	0.13 (0.09 to 0.18)	0.13 (0.09 to 0.18)
White Matter CVR Magnitude %/mmHg, median (IQR), in n = 47	0.05 (0.04 to 0.08)	0.05 (0.04 to 0.08)

CRAE: central retinal artery equivalent; CRVE: central retinal vein equivalent; AVR: artery/vein ratio; ΔCRAE: change in CRAE with CO_2_: ΔCRVE: change in CRVE with CO_2_: ΔAVR: change in AVR with CO_2_.

a n = 47 for the final two rows.

### Study size

Our sample was opportunistic, since a key aim of this pilot study was to assess feasibility of retinovascular reactivity in people with cSVD. Participants were recruited from an MRI study^5^ which had a target sample of 54 participants, based on an expected difference in CVR magnitude between participants with mild and severe WMH of 25%, accounting for 90% MRI completion rate (n = 54 is 90% of 60).

### Statistical methods

Statistical analysis was done using R version 3.3.0 after completion of all image processing and after the dataset was locked. We analysed data on a complete case basis. Distributions of variables and pairwise associations were assessed visually using relevant graphs. Variables with severely skewed distributions were log-transformed to normal. Associations were estimated using linear regression or ordered logistic regression, depending on the nature of the dependent variable. All models including change in retinal vessel width (Δ) were adjusted for relevant baseline retinal values. We also adjusted for age and systolic blood pressure as potential confounders. We reviewed model assumptions by examining heteroscedasticity and normality of residuals, and checked variable inflation factors (limit of 3) to assess co-linearity. In addition to beta-coefficients (β) we report the coefficient of determination (R2), which indicates the proportion of variance in the dependent variable accounted for by the independent variables, as well as root mean squared error (RMSE) and Akaike Information Criterion (AIC), to allow comparison between models. Our analysis of associations between retina and brain is exploratory rather than to test hypotheses about the general cSVD population from our sample, and so we did not adjust for multiple hypotheses^46^ and primarily report effect sizes and 95% confidence intervals (CI) to suggest magnitude of associations. However, we note that 95%CI excluding 0 indicate p < 0.05. We assessed agreement between repeat retinal images using Bland-Altman plots and repeatability coefficients, which indicate the estimated upper limit of difference between repeated measurements in 95% of cases.^47^

## Results

Of 60 participants 53 had structural MRI. All 53 also had usable baseline retinal imaging (breathing air). Of these, 47 also had CVR measured with BOLD MRI (Figure 1). Of the 53 participants with retinal imaging breathing air, 48 had useable fundus images during CO_2_ inhalation (Figure 1). Of these 48, 44 participants had useable CVR data. Figure 2 shows the sample derivation, and Table 1 the demographics for participants with and without retinal and brain imaging. Fig S1 shows Bland-Altman plots of AVR, CRAE, and CRVE in 8 participants with repeat retinal imaging on air. Repeatability coefficient for AVR was 0.09; CRAE 2.9 pixels; CRVE 4.5 pixels. For reference, the standard deviation of change with CO2 was: ΔAVR 0.09; ΔCRAE 2.1 pixels; ΔCRVE 4.8 pixels. Therefore, repeatability of AVR and CRVE was ≤1 standard deviation of ΔAVR and ΔCRVE, respectively.

At baseline breathing air, median (IQR) CRAE was 30.7 (28.1 to 32.6) pixels, CRVE was 40.4 (37.1 to 43.0) pixels, and AVR was 0.75 (0.7 to 0.8) (Table 1). These had weak associations with the severity of WMH (p > 0.05, Fig S2).

### Change in retinal vessel widths with CO_2_ is distributed around zero

**Figure 3.**
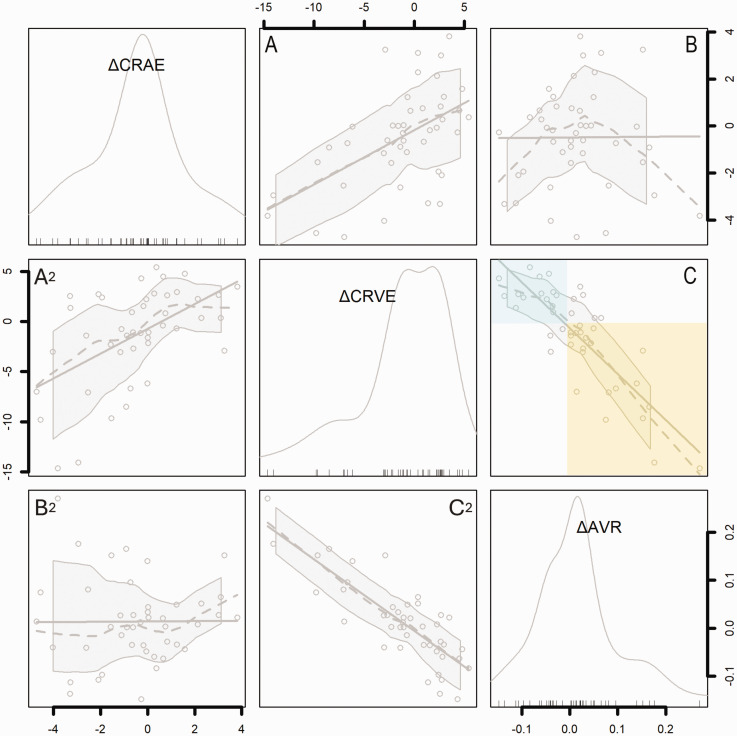
Correlogram showing the contributions of changes in arterioles (ΔCRAE) and venules (ΔCRVE) to change in the artery/vein ratio (ΔAVR). Histograms show the distribution of each variable, and axes indicate corresponding values within histograms in pixels (ΔCRAE, ΔCRVE) or pixel ratio (ΔAVR). Scatterplots show associations between ΔCRAE, ΔCRVE, and ΔAVR. Dotted lines show local regression (LOESS) with shaded 95% confidence intervals. Solid lines show linear regression. Note that each pairwise association between the three variables is illustrated by two scatterplots which have opposite X and Y axes (therefore A2 is the mirror image of A, etc.). Top left histogram: ΔCRAE is distributed around zero (median −0.3, range −4.7 to 3.8); Middle histogram: ΔCRVE has a marked negative skew (median −0.7, range −14.6 to 5.4); Bottom right histogram: ΔAVR has a positive skew (median 0.01, range −0.15 to 0.27). (a) ΔCRVE is associated with ΔCRAE (r 0.52, p<0.01). (b) ΔAVR is not associated with ΔCRAE (r 0.03, p=0.82) and (c) ΔAVR is strongly associated with ΔCRVE (r −0.82, p<0.01). Note that ΔAVR greater than zero is related to negative ΔCRVE, i.e., narrowing of venules with CO_2_ (yellow box), while ΔAVR less than zero is related to widening of venules with CO_2_ (blue box).

Figure 3 shows the change in retinal arterial width, venous width, and artery-vein ratio in response to CO_2_ (ΔCRAE, ΔCRVE, ΔAVR, respectively). Fig S3 shows group and individual level changes in retinal vessel measurements. ΔCRAE and ΔAVR had average values close to zero and scatter in positive and negative directions, and ΔCRVE had a notably negative skew. In our sample ΔAVR was associated with ΔCRVE, but not ΔCRAE (Figure 3(b) and (c)). An increase in AVR with CO_2_ (e.g., 0 to 0.2; i.e., a positive ΔAVR) was mainly attributable to venular narrowing (Figure 3(b), yellow box) while a decrease in AVR (e.g. 0 to −0.15; i.e., a negative ΔAVR) was mainly attributable to venular widening (blue box). ΔCRAE and ΔCRVE were positively correlated with each other such that widening or narrowing of arterioles tended to correspond to a similar direction of calibre change in venules (Figure 3(a)), although the magnitude of arteriolar change was less than that of venules.

### ΔAVR is inversely associated with white matter hyperintensities

**Figure 4.**
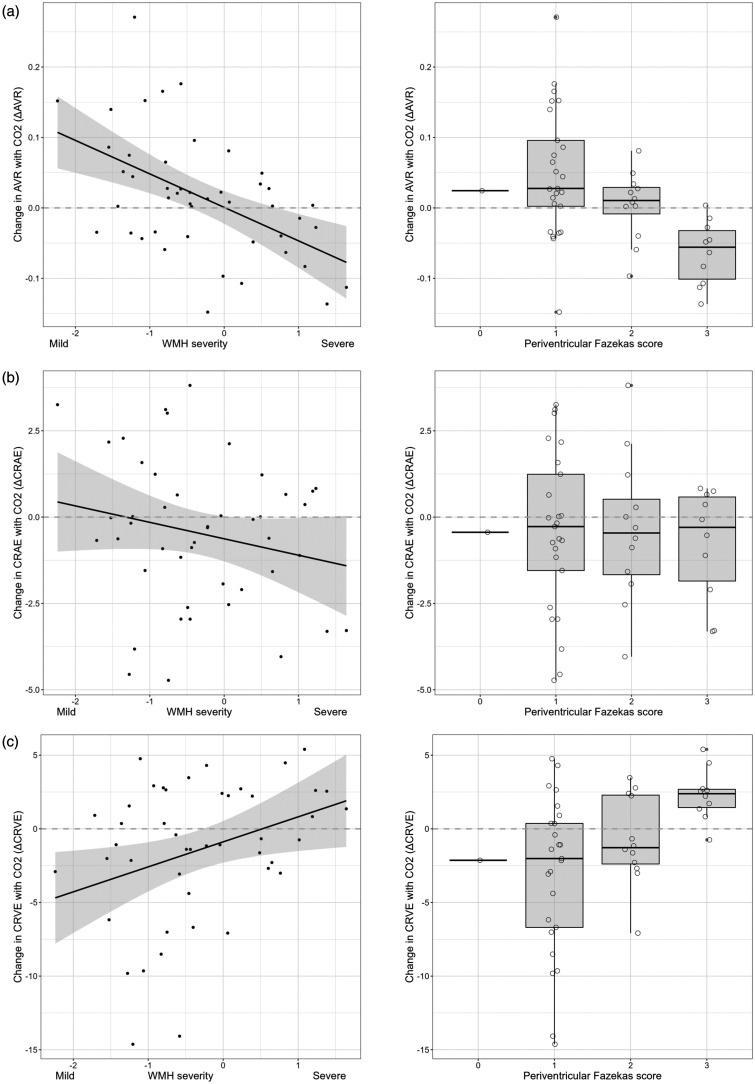
Change in artery/vein ratio with CO_2_ (ΔAVR) against severity of while matter hyperintensities (WMH) in cSVD, in whole brain and periventricular white matter. (a) Association with WMH volume, line of best fit with shaded 95% confidence intervals, and periventricular Fazekas WMH score excluding one subject with score = 0. (b) ΔCRAE against WMH volume and periventricular Fazekas WMH score and (c) ΔCRVE against WMH volume and periventricular Fazekas WMH score. See Table S1 for details of models adjusted only for baseline retinal values. See Table 2 for models additionally adjusted for age and systolic blood pressure.

The volume of WMH, log-normalised to intracranial volume, was inversely correlated with ΔAVR, such that greater volume of WMH was associated with a decrease in AVR in response to CO_2_ (a negative ΔAVR) and a smaller volume of WMH was associated with a positive ΔAVR (adjusted R2 0.28, β −6.4 (95%CI −9.3 to −3.5)). There were similar associations for Fazekas scores (Figure 4(a), Table S1). Table 2 shows these associations, adjusted for age and systolic blood pressure as well as baseline AVR. Notably, the coefficient of determination for ΔAVR against WMH, adjusted for baseline AVR, age, and systolic blood pressure (adjusted R2 0.47, RMSE 0.64) suggests that these variables account for nearly half the variance in WMH severity in our sample (Table 2). For context, this is greater than the association between white matter CVR and WMH within the same person’s brain, adjusted for age and systolic blood pressure (adjusted R2 0.38, RMSE 0.71, β −10.2 (−18.6 to −1.8)).

**Table 2.** Associations between change in retinal vessel widths, white matter hyperintensities, and cerebrovascular reactivity in subcortical grey matter and cerebral white matter.

Brain variable	Retinal variable	N	Coefficient(β or log odds)	2.5% CI	97.5% CI	Adjusted R2	RMSE	AIC
WMH	ΔAVR	47	−5.22	−**7.99**	−**2.45**	0.47	0.64	30.7
Total Fazekas	ΔAVR	47	−11.19	−**19.52**	−**2.85**	–	–	146.9
PVWM Fazekas	ΔAVR	48	−24.49	−**40.81**	−**8.17**	–	–	77.1
Deep Fazekas	ΔAVR	43	−23.92	−**39.73**	−**8.09**	–		75.5
CVR GM	ΔAVR	44	0.29	**0.06**	**0.54**	0.21	0.05	−245.3
CVR WM	ΔAVR	44	0.15	**0.04**	**0.25**	0.21	0.02	−317.9
WMH	ΔCRVE	47	0.04	−0.02	0.09	0.35	0.71	−21.2
Total Fazekas	ΔCRVE	47	0.09	−0.04	0.23	–	–	161.5
PVWM Fazekas	ΔCRVE	48	0.20	−0.01	0.41	–	–	95.7
Deep Fazekas	ΔCRVE	43	0.14	−0.04	0.32	–	–	122.8
CVR GM	ΔCRVE	44	−0.01	−0.01	0.00	0.17	0.06	−243.4
CVR WM	ΔCRVE	44	−0.01	−**0.01**	−**0.00**	0.19	0.02	−316.5
WMH	ΔCRAE	47	−0.09	−0.19	0.02	0.40	0.68	−24.9
Total Fazekas	ΔCRAE	47	−0.13	−0.38	0.12	–	–	160.3
PVWM Fazekas	ΔCRAE	48	−0.05	−0.34	0.23	–	–	97.6
Deep Fazekas	ΔCRAE	43	−0.08	−0.35	0.19	–	–	125.1
CVR GM	ΔCRAE	44	−0.00	−0.01	0.01	0.10	0.06	−239.8
CVR WM	ΔCRAE	44	−0.00	−0.01	0.00	0.07	0.03	−310.3

Associations are adjusted for age, systolic blood pressure, and baseline values of retinal change variables. Models were assessed for variable inflation (VIF < 3), normality of residuals (QQ plots, Shapiro-Wilk test p > 0.05), and heteroskedasticity (OLS score test p > 0.05). Continuous white matter hyperintensity and cerebrovascular reactivity values were analysed with linear regression (coefficient: β); Fazekas scores were analysed with ordered logistic regression excluding participants with scores = 0 (coefficient: ordered log odds). Bold text indicates coefficients with 95% confidence intervals that do not cross zero, indicating a conventional threshold of p < 0.05. ΔAVR: change in AVR with CO_2_; ΔCRVE: change in CRVE with CO_2_; ΔCRAE: Change in CRAE with CO_2_; WMH: white matter hyperintensity volume: log normalised by intracranial volume; Total Fazekas: score for whole brain; PVWM Fazekas: score for periventricular white matter; Deep Fazekas: score for deep white matter; CVR GM: cerebrovascular reactivity in subcortical grey matter; CVR WM: cerebrovascular reactivity in white matter; n: sample size; β: beta-coefficient (linear slope in units of x and y variables); 2.5% and 97.5% CI: low and high limits of 95% confidence intervals respectively; adjusted R2: adjusted multiple coefficient of determination; RMSE: root mean-squared error; AIC: Akaike information criterion.

ΔCRVE was also associated with WMH (overall volume, periventricular and deep Fazekas scores) (Figure 4(c), Table S1), but not after adjusting for age and systolic blood pressure (Table 2).

ΔCRAE did not have a pairwise association with WMH for the sample as a whole. However, since AVR mathematically combines CRAE and CRVE, individual-level arteriole responses to CO_2_ may explain why ΔAVR is associated with WMH more strongly than ΔCRVE alone (Table 2). Consistent with this, a model of WMH including ΔCRAE and ΔCRVE as separate terms, instead of ΔAVR, suggested that changes in both arterioles and venules with CO_2_ may be associated with severity of WMH when adjusted for each other and baseline values. In this model, ΔCRAE was inversely associated with WMH (β −0.3), and ΔCRVE directly associated with WMH (β 0.1) (p < 0.05 for each) (Table S2).

### ΔAVR is directly associated with CVR in white matter and deep grey matter

**Figure 5.**
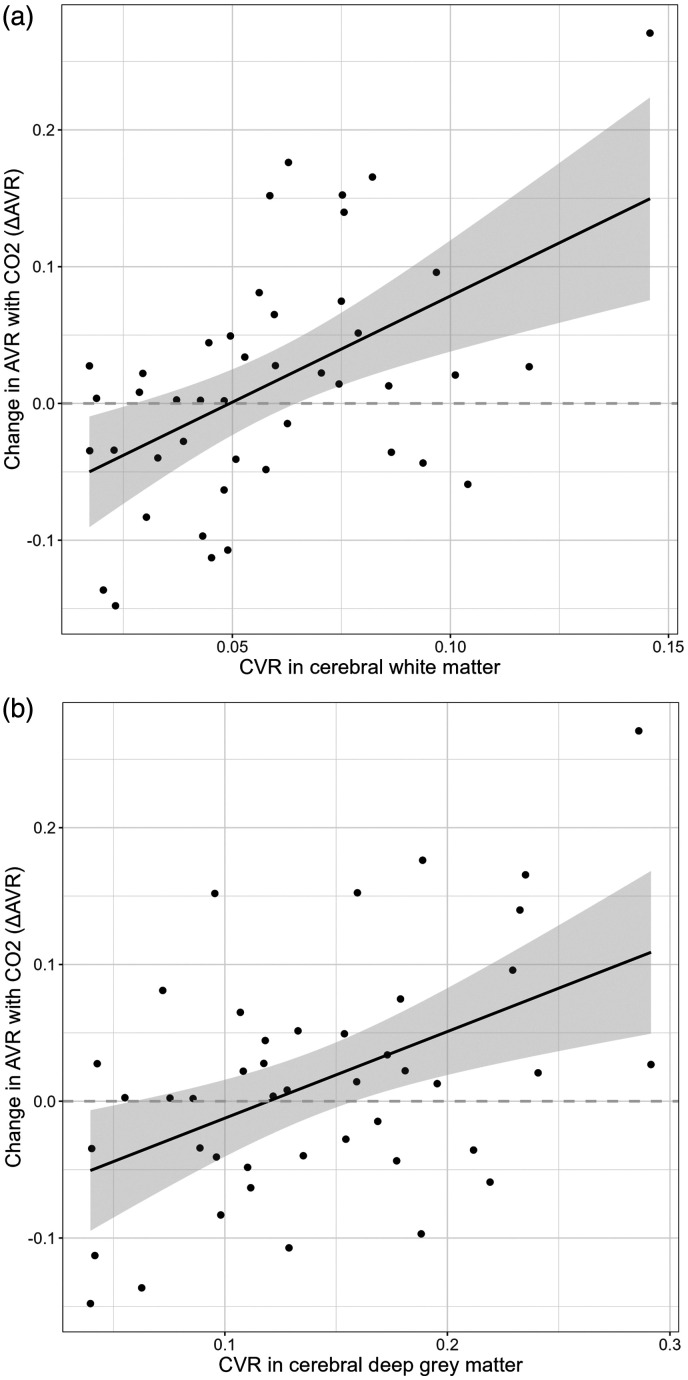
Correlation between change in artery/vein ratio with CO_2_ (ΔAVR) and cerebrovascular reactivity (CVR) to 6% CO_2_ challenge. Line of best fit with shaded 95% confidence intervals. See Table S1 for details of models adjusted only for baseline retinal values, and Table 2 for models additionally adjusted for age and systolic blood pressure. (a) Cerebral white matter and (b) deep grey matter.

Associations between ΔAVR and CVR in white and deep grey matter are shown in Figure 5. These correspond to regression coefficients: adjusted R2 0.21, β 0.15 (0.04 to 0.25), and adjusted R2 0.21, β 0.29 (0.06 to 0.54) for white and deep grey matter respectively (Table 2, Table S1). ΔCRVE was moderately and inversely correlated with CVR in white and deep grey matter (Table 2), consistent with the interpretation that changes in venous width are the main influence on the association between ΔAVR and CVR in these brain regions. Analysis of ΔCRAE and ΔCRVE as separate variables within models of CVR is in Table S2.

### Pairwise associations between retinal vessel metrics and other variables

ΔAVR was moderately correlated with baseline CRVE (Pearson r 0.38), such that relatively wide venules at baseline were more likely to narrow with inhaled CO_2_, compared to venules with a narrower calibre at baseline. A similar correlation was seen between ΔAVR and mean cell volume (r 0.43), but not haematocrit. Systolic blood pressure, measured throughout imaging, was inversely correlated with ΔAVR (r −0.42), so that people with higher blood pressure were more likely to have a negative ΔAVR response to CO_2_. A range of exploratory pairwise correlations with retinal variables are summarised in Fig S2. Demographic and imaging variables are compared between participants with negative or positive change in ΔAVR, and ΔCRVE in Table S4, A and B respectively. Narrowing of retinal venules to CO_2_ was associated with lower alcohol intake, lower systolic blood pressure, and longer time between index stroke and recruitment to our study (Table S4 B).

## Discussion

We measured retinal vessel reactivity to inhaled CO_2_ (6% in air) in people with cSVD to investigate feasibility and associations with severity of cSVD features and CVR. We found substantial associations between retinal vessel reactions to CO_2_, WMH, and vessel reactivity in the brain (CVR) in people with cSVD. The strongest of these appear to be between ΔAVR, the severity of WMH, and CVR in white or deep grey matter (adjusted R2 0.47, 0.21 and 0.21 respectively). CVR measured with BOLD MRI is known to be inversely associated with WMH severity^6,11^ and in our sample, white matter CVR and ΔAVR both had comparably strong negative associations with WMH (adjusted R2 0.38 and 0.47 respectively). We found that greater severity of WMH was associated with a decrease in AVR with CO_2_ (ΔAVR <0), and that milder WMH was associated with an increase in AVR with CO_2_ (ΔAVR >0) (Figure 4(a)). We also found that ΔAVR was directly associated with CVR, consistent with links between milder WMH, greater ΔAVR, and greater CVR.

AVR is a ratio of arteriole to venule width, and therefore AVR (and ΔAVR) results from combinations of relative changes in arterioles and venules within individuals. This is illustrated in Table S3. In our sample ΔAVR appears to have been driven chiefly by changes in venules. This can be seen from the strong association between ΔCRVE and ΔAVR, in contrast to the absence of a clear group-level association between ΔCRAE and ΔAVR (Figure 3). In addition, ΔAVR was associated with severity of WMH (Figure 4(a), Table 2), similar to a model of WMH including ΔCRAE and ΔCRVE as separate terms (Table S2). This suggests that people with worse WMH do not simply have lower overall reactivity of microvessels, but dysfunctional coordination of arteriolar and venular diameters, with relative widening of venules and (less obviously) individual-level narrowing of arterioles in response to 6% CO_2_. This is illustrated by negative β for ΔCRAE, and positive β for ΔCRVE, in Table S2. While ΔAVR captures these individual-level relationships, in isolation it cannot explain the exact arteriolar or venular contributions, hence we have also unpicked the different combinations that can lead to positive or negative ΔAVR (Table S3).

Unlike retinal imaging, BOLD MRI cannot distinguish between arteriolar, capillary or venular contributions to microvascular blood flow since it estimates CVR via blood oxygenation across voxels containing many small vessels. In contrast, colour fundus images capture the widths of distinct vessel segments measuring ∼100–150 μm at the optic disc – similar to larger vessels penetrating cerebral white matter.^13^ Newer ophthalmic imaging modalities, such as adaptive optics, can measure capillary diameters (∼5 μm) and perhaps reactive changes in capillary width.^48,49^ Retinal imaging may provide the opportunity to compare the effects of challenges such as inhaled CO_2_ on different points in the microvascular tree. Our data, combined with the biologically plausible assumption that inhaled CO_2_ acts on retinal and cerebral vessels in similar ways and to a similar extent,^16,17^ suggest that the severity of WMH in cSVD may be associated with effects on the venular side of the circulation as well as on the arteriolar side, and abnormal arteriovenous coordination.

Our finding of widening of venules (and negative ΔAVR) associated with more severe WMH adds to previously reported associations with *static* retinal vessel measurements. *Static* fundus imaging shows that retinal venules are wider in people with lacunar stroke and WMH, compared to other types of ischaemic stroke, in cross-sectional studies.^50–52^ Larger CRVE is also associated with greater risk of cSVD progression over time.^26^ Clusters of widened cerebral vessels within WMH have been observed on 3 T susceptibility-weighted MRI in patients with severe WMH, and were related to reduced CVR,^53^ may relate to local WMH cavitation, and could represent abnormally widened *static* cerebral microvessels as a possible localised cerebral corollary of the generally widened retinal venules seen in lacunar stroke on static retinal imaging. Venules that react by widening in response to inhaled CO_2_ could theoretically reflect arteriolar-to-venular shunting with blood rapidly reaching the venules by bypassing the capillary bed owing to misdirected arteriole-to-capillary flow control, and reduced transit-time of erythrocytes perfusing these regions - contributing to ischaemia by exacerbating mismatch of local metabolism and perfusion.^54,55^ A dysfunctional state is further supported by the finding of narrower baseline venule calibre being more likely to widen with CO_2_, (r −0.46, Fig S2) and higher systolic BP being more likely to have negative ΔAVR (i.e., more venular widening) with CO_2_ (r −0.44, Fig S2).

On the other hand, we observed narrowing of venules and (less convincingly) of arterioles in response to CO_2_, associated with milder WMH. Narrowing of venules was particularly marked in some cases (as much as −15 pixels) (Figure 3). This contrasts with data from healthy young people, in whom inhaled CO_2_ causes average widening of both retinal arterioles and venules^20^ and increased blood flow^56^ thought to be mediated by H^+^ and prostaglandins.^17^ Similarly, the normal retinal neurovascular coupling response to light involves widening of arterioles and venules. In healthy young people this is blunted by inhibiting nitric oxide synthase with L-NMMA,^57^ although the modulatory role of NO on retinal neurovascular coupling appears to be more complex in models of diabetic retinopathy.^58^ Narrowing of retinal venules could have several explanations.

One explanation is that venules might merely be measured more accurately than arterioles, since venules are larger. However, repeatability coefficients were reasonable in eight participants with repeated good-quality baseline images (Fig S1), suggesting changes are detectable in both vessel types. A larger sample would have more power to detect small but important changes in CRAE that did not reach significance in our small sample.

Another explanation is that venular narrowing resulted from increased blood volume retained in arterioles during initial arteriole dilation caused by CO_2_.^20^ This is consistent with our observation of venule narrowing in the context of positive ΔAVR, which could result from relative arteriolar widening, venular narrowing, or combination of both (Table S3).

It is also possible that venule narrowing indicates active constriction. Venules are traditionally regarded as passive vessels, and the walls of first-order retinal venules are thin with only sparse smooth muscle.^59^ However, retinal venules contain contractile pericytes^60,61^ and isolated ex-vivo porcine retinal venules constrict in response to endothelin 1 and dilate in response to adenosine.^48,59,62^ This suggests that healthy retinal venules may have some inherent ability to react, which could be lost in microvascular disease. In theory, venular constriction could help ameliorate metabolism/perfusion mismatch by increasing capillary transit-time.^54,55^ This would be consistent with associations between positive ΔAVR and venule narrowing, with better CVR and less severe WMH. It could also be consistent with associations between venule narrowing and longer time from index stroke, lower systolic blood pressure, and lower alcohol intake (Table S4 B)

We cannot be sure of the reason for the observed changes in venular calibre. However, small reductions in venous diameter can contribute to a partial uncoupling of capillary pressure and flow via hemorheological effects,^63^ and haematological variables such as haematocrit could also contribute to retinal and cerebrovascular calibre and reactivity.^64^ Haematocrit was not strongly associated with vessel widths in our sample (Fig S2), but it is possible that it and related haematological variables, such as mean cell volume, could have a combined effect on small vessel rheology. Future studies of vascular reactivity in cSVD may benefit from measuring hemorheological variables in larger groups.

Our results suggest that larger studies are warranted since we found the technique is feasible in people with cSVD, and it may reveal information about microvascular coordination that is related to WMH. Flicker-induced dilation is an alternative method to CO_2_ reactivity, which may be more practical. It tests neurovascular coupling^57^ rather than chemo-regulation. Flicker-induced dilation has been suggested as a proxy for endothelial dysfunction in cardiovascular disease, but again there are few data from people with cerebrovascular disease.^30,34^

Our study has limitations. It is exploratory, with a relatively small sample from one site, and the associations we report require confirmation in further clinical studies. We performed only limited statistical adjustments and cannot comment on the roles of a range of potential confounders besides age and systolic blood pressure. We note however that our current aim is only to explore the existence of associations between retinovascular reactivity and brain imaging in cSVD, and not to determine the precise mechanisms behind any associations. That objective will require further clinical studies with appropriate design and sample size, and we hope that our present study will help motivate that future work. We did not find obvious associations between baseline static CRAE, CRVE, or AVR and WMH (Fig S2), likely due to our small sample. Adequate retinal imaging was possible in most participants (73.3%), but quality was poorer when participants were breathing CO_2_, likely due to head movement, in addition to general challenges of fundus photography through undilated pupils. Image quality could be improved in future by dilating pupils before imaging and using a smaller face mask. We compared retinal vessel width in retinal images against CVR measured by BOLD MRI, effectively using both vessel width and blood oxygenation as proxies for microvascular blood flow in different parts of the central nervous system, and the CO_2_ protocols for retina and brain imaging were not identical. The CVR metric is a ratio incorporating an adjustment for EtCO_2_, while our retinal vessel variables describing the response to CO_2_ are differences (i.e., subtracted rather than divided) and were not adjusted for EtCO_2_. Future work might attempt to compare retinal and cerebral blood flow by using methods such as laser doppler flowmetry, analyse EtCO_2_-adjusted ratio metrics for both retina and brain, use identical CO_2_ protocols for retina and brain imaging, or use visual stimuli to trigger reactive hyperaemia in retina and visual cortex rather than CO_2_ to provoke systemic vasodilation. We also note that unlike the brain, the retinal microvasculature does not have autonomic innervation distal to the central retinal artery.^17^ This could explain differences between vessel reactivity in retina and brain. Future work could include imaging of the choroidal circulation (lying between the retina and sclera), since this does have autonomic innervation,^17^ and a comparison of choroidal with retinal and cerebrovascular reactivity could be informative.

In conclusion, measuring retinal vessel reactivity to CO_2_ is feasible in people with cSVD and appears to have strong associations with white matter disease, comparable to those between WMH and CVR measured with BOLD MRI. Venules may have an active role in the relationship between lower CVR and higher WMH, and our observation of venular widening in participants with more severe cSVD, and narrowing in those with less severe cSVD, may suggest a microvascular function ‘tipping point’ in the trajectory of cSVD. Our data begin to address an important gap in understanding about relationships between dynamic microvascular function across retina and brain in people with established cSVD. We suggest that retinal imaging may be a relatively accessible and inexpensive method for studying neurovascular reactivity in people with cSVD.

### What is the current knowledge on the topic?

- Cerebral small vessel disease leads to stroke and dementia
- Cerebrovascular reactivity declines with increasing severity of cerebral small vessel disease
- It is not possible to discern the individual contribution of cerebral arterioles and venules to cerebrovascular reactivity

### What question did this study address?

- Is retinal vessel reactivity feasible in people with cerebral small vessel disease?
- If so, is it associated with white matter hyperintensity volume and cerebrovascular reactivity?

### What does this study add?

- Retinal vessel reactivity is associated with white matter hyperintensity volume and cerebrovascular reactivity in cerebral small vessel disease
- Differential arteriole and venule responses to CO2 challenge suggests shunting might bypass capillaries

### How might this potentially impact on the practice of neurology?

- Retinal vessel reactivity may be a practical way to measure the coordination of central nervous system arteriole and venule reactivity in people with cerebral small vessel disease

## Supplemental Material

sj-pdf-1-jcb-10.1177_0271678X251366079 - Supplemental material for Retinal vascular reactivity is associated with white matter hyperintensities and dysfunctional cerebrovascular reactivity in cerebral small vessel disease Supplemental material, sj-pdf-1-jcb-10.1177_0271678X251366079 for Retinal vascular reactivity is associated with white matter hyperintensities and dysfunctional cerebrovascular reactivity in cerebral small vessel disease by Gordon W Blair, Ian J C MacCormick, Sarah McGrory, Tom MacGillivray, Iona Hamilton, Yulu Shi, Francesca Chappell, Michael J Thrippleton, Michael S Stringer, Fergus Doubal and Joanna M Wardlaw in Journal of Cerebral Blood Flow & Metabolism

## Data Availability

The full individual patient data anonymized data set, along with the study protocol, is available to bona fide researchers via the University of Edinburgh's Cerebrovascular Diseases Database. Access request should be submitted to J.M. Wardlaw along with a description of any planned analyses.
