# Supplementary material for: Retinal vascular reactivity is associated with white matter hyperintensities and dysfunctional cerebrovascular reactivity in cerebral small vessel disease
Source: J Cereb Blood Flow Metab. 2025 Aug 6:0271678X251366079. Online ahead of print. doi: 10.1177/0271678X251366079 (PMC12331647; doi:10.1177/0271678X251366079)
Supplement: sj-pdf-1-jcb-10.1177_0271678X251366079 - Supplemental material for Retinal vascular reactivity is associated with white matter hyperintensities and dysfunctional cerebrovascular reactivity in cerebral small vessel disease [file sj-pdf-1-jcb-10.1177_0271678X251366079.pdf]

Supplementary material

Figure S1) Bland-Altman plots showing variation within eyes from 8 participants with repeated good quality retinal images taken breathing air. Dashed lines indicate mean difference between images and two standard deviations from the mean difference. Scatter plots showing correlation between repeated measurements in the same participants, with line of best fit (dashed) and 95% confidence intervals (shaded area), and line of perfect agreement (solid line). A) artery-vein ratio; B) Central retinal artery equivalent; C) Central retinal vein equivalent.

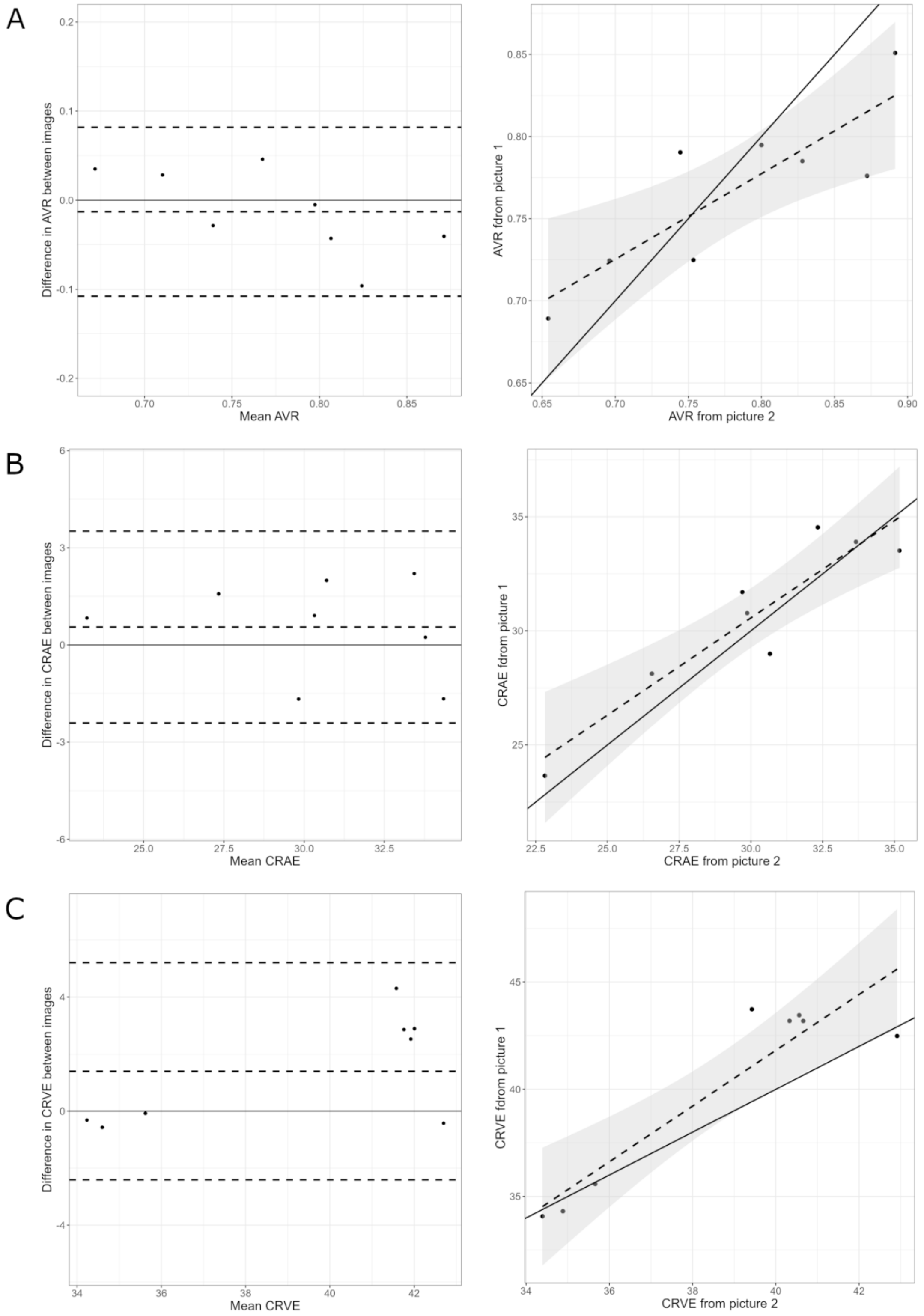

Figure S2 A) Pairwise Pearson correlations between retinal variables and systemic variables. CRAE, central retinal artery equivalent; CRVE, central retinal vein equivalent; AVR, artery-vein ratio; ΔCRAE, change in CRAE with CO_2; ΔCRVE, change in CRVE with CO_2; ΔAVR, change in AVR with CO_2; log WMH volume, log-transformed volume of white matter hyperintensities, adjusted for intracranial volume; CVR, cerebrovascular reactivity; BP, blood pressure.

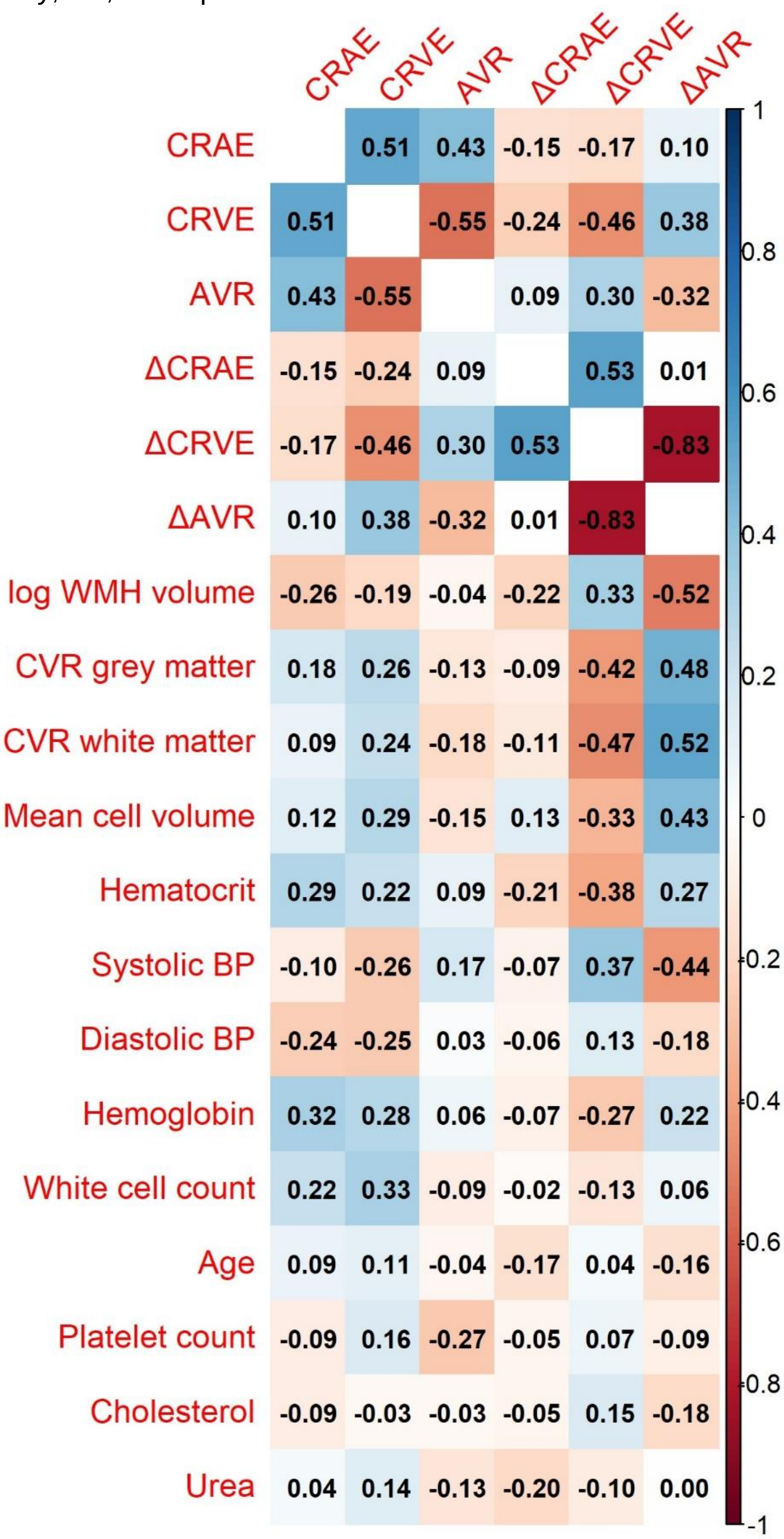

Figure S2 B) Pairwise Pearson correlations between retinal variables and additional MRI variables. Total arterial flow (ml/min/100ml tissue); Int carotid PI, Internal carotid pulsatility index; Sup sag sinus PI, Superior sagittal sinus pulsatility index; Trans sinus PI, Transverse sinus pulsatility index; Straight sinus PI, Straight sinus pulsatility index; Aqueduct CSF flow (ml/min); Aqueduct stroke volume (ml); Foramen magnum CSF flow (ml/min); Foramen magnum stroke volume (ml); CVR delay, cerebrovascular reactivity delay (seconds); PVS, perivascular space score (1-4).

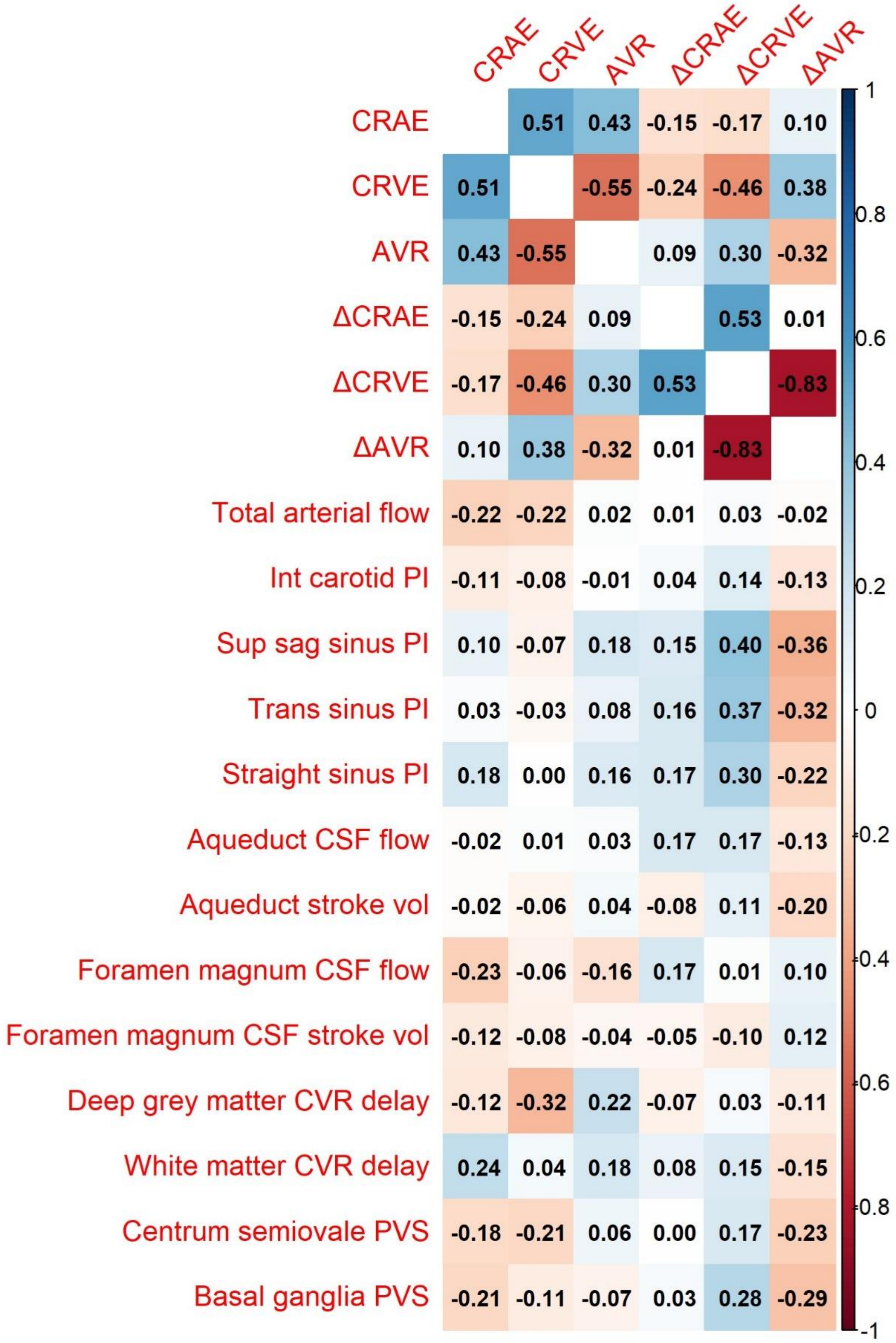

Figure S3 A) Central retinal artery equivalent (CRAE) while breathing air, and 6% CO_2 in air. Boxes show median and inter-quartile range.

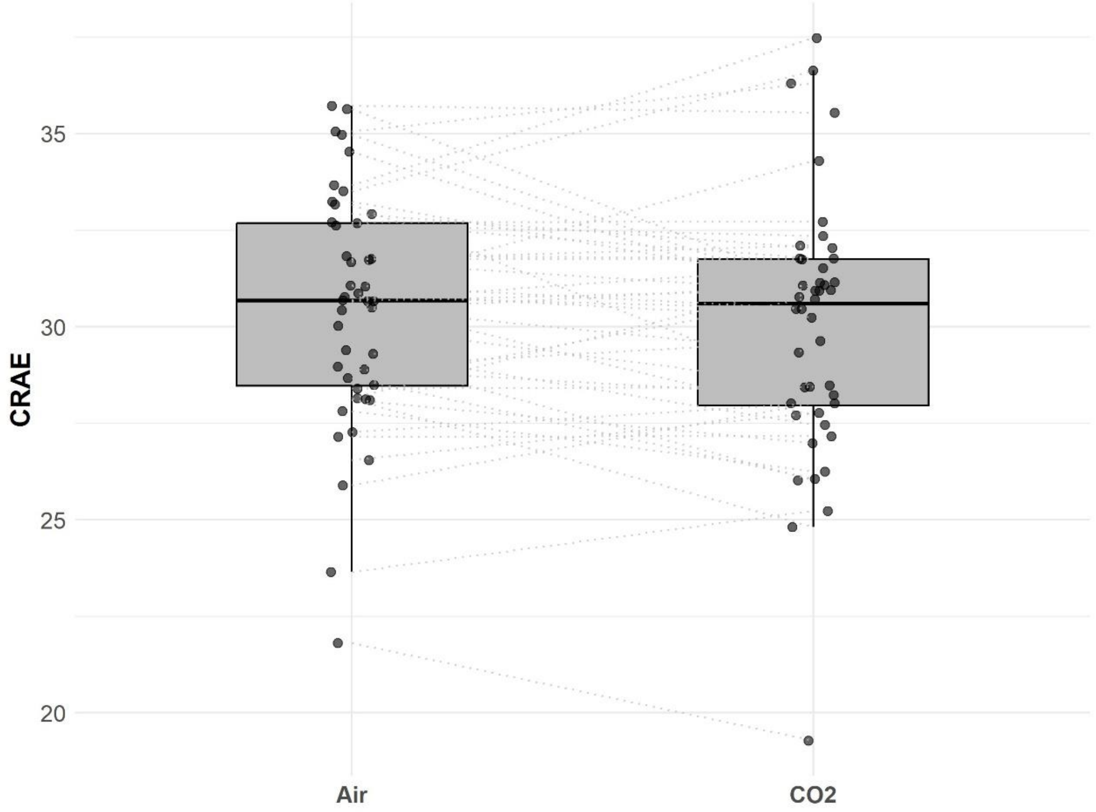

Figure S3 B) Central retinal vein equivalent (CRVE) while breathing air, and 6% CO_2 in air. Boxes show median and inter-quartile range.

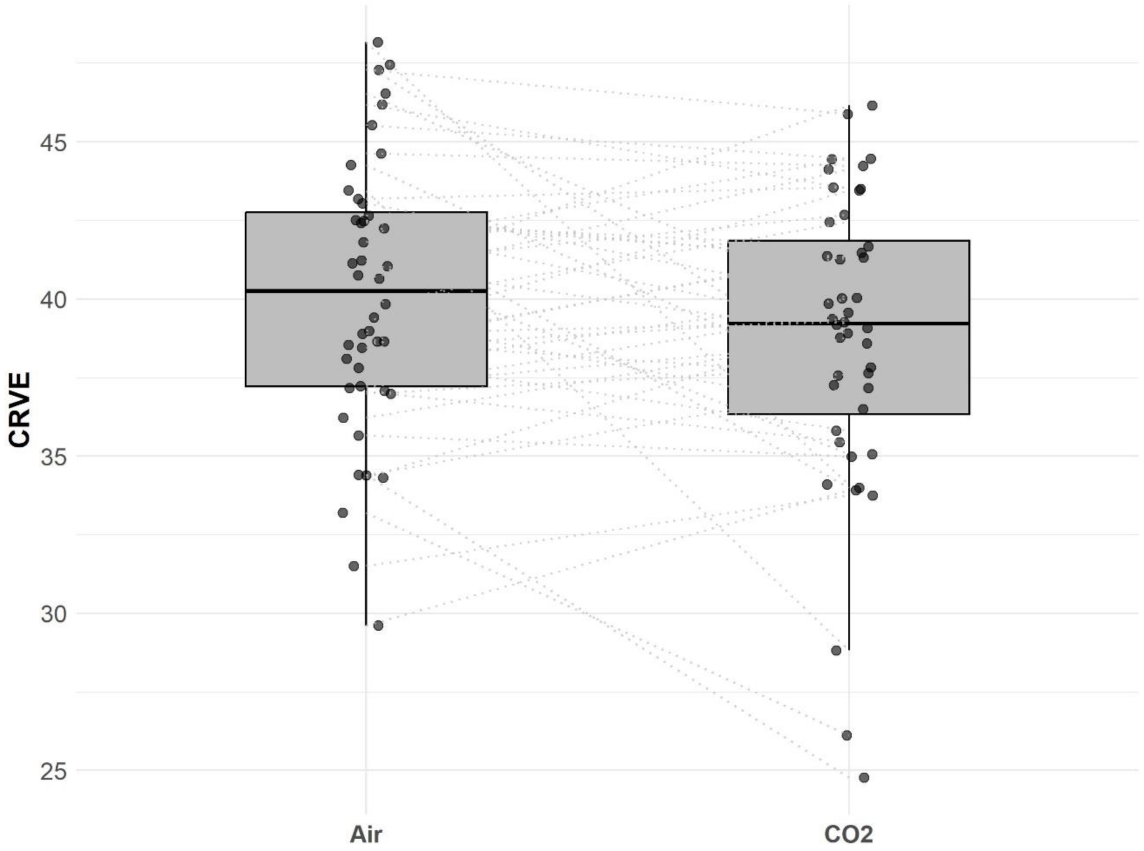

Figure S3 C) Artery-vein ratio (AVR) while breathing air, and 6% CO_2 in air. Boxes show median and inter-quartile range.

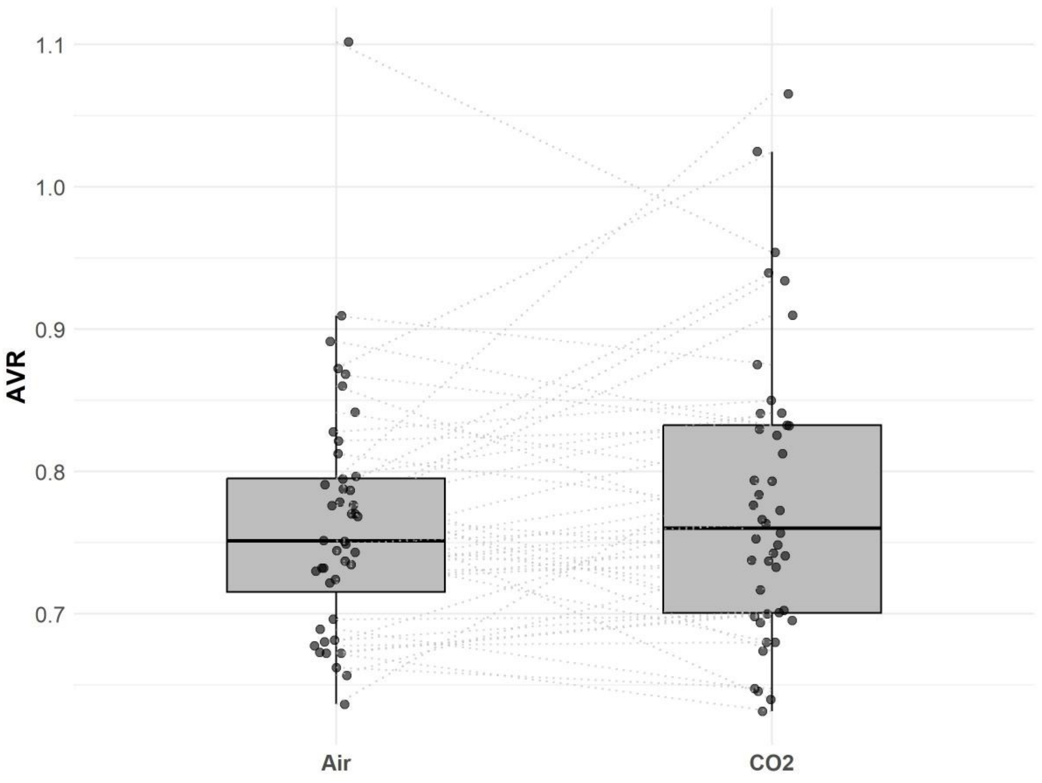

1.1
1.0
0.9
0.8
0.7
AVR
Air
CO2

Supplementary tables

Table S1) Associations between change in retinal vessel widths, white matter hyperintensities, and cerebrovascular reactivity in deep grey matter and cerebral white matter. These are adjusted only for baseline values of retinal change variables (i.e., baseline AVR, CRVE, CRAE). Bold indicates $p<0.05$. Models were assessed for variable inflation (VIF<3), normality of residuals (QQ plots, Shapiro-Wilk test $p>0.05$), and heteroskedasticity (OLS score test $p>0.05$).

Brain variable	Retina variable	N	Coefficient (β or log odds)	2.5% CI	97.5% CI	Adjusted R2	RMSE	AIC
WMH	ΔAVR	47	-6.4	**-9.33**	**-3.48**	0.28	0.77	-18.4
Total Fazekas	ΔAVR	47	-12.79	**-20.43**	**-5.14**	-	-	153.5
PVWM Fazekas	ΔAVR	48	-23.80	**-37.51**	**-10.09**	-	-	83.7
Deep Fazekas	ΔAVR	43	-24.16	**-38.24**	**-10.09**	-	-	75.2
CVR GM	ΔAVR	44	0.36	**0.14**	**0.58**	0.19	0.06	-246.2
CVR WM	ΔAVR	44	0.17	**0.08**	**0.26**	0.23	0.02	-320.5
WMH	ΔCRVE	47	0.06	-0.01	0.12	0.08	0.87	-7.1
Total Fazekas	ΔCRVE	47	0.11	-0.01	0.24	-	-	171.0
PVWM Fazekas	ΔCRVE	48	0.23	**0.05**	**0.41**	-	-	103.9
Deep Fazekas	ΔCRVE	43	0.18	**0.02**	**0.35**	-	-	121.3
CVR GM	ΔCRVE	44	-0.01	**-0.01**	**-0.00**	0.15	0.06	-244.0
CVR WM*	ΔCRVE	44	-0.00	**-0.00**	**-0.00**	0.19	0.03	-318.1
WMH	ΔCRAE	47	-0.12	-0.25	0.01	0.12	0.85	-8.9
Total Fazekas	ΔCRAE	47	-0.14	-0.39	0.10	-	-	172.5
PVWM Fazekas	ΔCRAE	48	-0.08	-0.35	0.19	-	-	111.7
Deep Fazekas	ΔCRAE	43	-0.1	-0.36	0.17	-	-	126.1
CVR GM	ΔCRAE	44	-0.00	-0.01	0.01	-0.00	0.06	-236
CVR WM	ΔCRAE	44	-0.00	-0.01	0.00	-0.02	0.03	-308.2

*Shapiro-wilk normality test p=0.02.
Abbreviations: ΔAVR, change in AVR with CO_2; ΔCRVE, change in CRVE with CO_2; ΔCRAE, Change in CRVE with CO_2; WMH, white matter hyperintensity volume, log normalised by intracranial volume; Total Fazekas, score for whole brain; PVWM Fazekas, score for periventricular white matter; Deep Fazekas, score for deep white matter; CVR GM, cerebrovascular reactivity in grey matter; CVR WM, cerebrovascular reactivity in white matter; n, sample size; β, beta-coefficient (linear slope in units of x and y variables); 2.5% and 97.5% CI, low and high limits of 95% confidence intervals respectively; adjusted R2, adjusted multiple coefficient of determination; RMSE, root mean-squared error; AIC, Akaike information criterion.

Table S2) Associations between retinal vessel response to CO_2, white matter hyperintensities (WMH), and cerebrovascular reactivity (CVR), with retinal arteriolar and venular response modelled as separate terms. Bold indicates p<0.05. Models are adjusted for baseline values for retinal vessel widths (CRAE and CRVE). Models were assessed for variable inflation (VIF<3), normality of residuals (QQ plots, Shapiro-Wilk test p>0.05), and heteroskedasticity (OLS score test p>0.05).

**Brain variable**	**Retinal variables**	**β Coefficient**	**2.5% CI**	**97.5% CI**	**Adjusted R2**	**RMSE**	**AIC**
WMH*	(Intercept)	1.99	-0.77	4.76	0.34	0.72	-20.3
	ΔCRAE	-0.25	**-0.38**	**-0.12**			
	ΔCRVE	0.12	**0.06**	**0.18**			
	Baseline CRAE	-0.10	**-0.19**	**-0.01**			
	Baseline CRVE	0.02	-0.05	0.09			
CVR GM	(Intercept)	0.05	-0.16	0.27	0.14	0.06	-241.7
	ΔCRAE	0.01	-0.00	0.02			
	ΔCRVE	-0.01	**-0.01**	**-0.00**			
	Baseline CRAE	0.00	-0.00	0.01			
	Baseline CRVE	0.00	-0.01	0.01			
CVR WM**	(Intercept)	0.04	-0.04	0.23	0.17	0.02	-315.3
	ΔCRAE	0.00	-0.00	0.01			
	ΔCRVE	-0.00	**-0.01**	**-0.00**			
	Baseline CRAE	0.00	-0.00	0.00			
	Baseline CRVE	0.00	-0.00	0.00			

*OLS score test p=0.05; **Shapiro-Wilk test p=0.03

Table S3) Illustration of the possible causes of decreased or increased artery/vein ratio (AVR) with CO_2 (ΔAVR). ΔAVR is defined as the difference in AVR breathing CO_2 and AVR breathing air. Because AVR is a ratio of artery and vein width, changes in AVR are the result of relative changes in artery and vein width. These are illustrated in (A to D).

A & B) A negative change in AVR with CO_2 (ΔAVR) can be caused by relative widening of veins, or relative narrowing of arteries, or a combination of both.

**A**	CO_2	Air	$\Delta AVR = \Delta AVR_{CO2} - \Delta AVR_{Air}$	Cause
Artery width	3	3	Negative ΔAVR	Artery constant with CO_2
Vein width	5	4		Vein wider with CO_2

**B**	CO_2	Air	$\Delta AVR = \Delta AVR_{CO2} - \Delta AVR_{Air}$	Cause
Artery width	2	3	Negative ΔAVR	Artery narrower with CO_2
Vein width	4	4		Vein constant with CO_2

C & D) A positive ΔAVR can be caused by a relative narrowing of veins, or relative widening of arteries, or a combination of both.

**C**	CO_2	Air	$\Delta AVR = \Delta AVR_{CO2} - \Delta AVR_{Air}$	Cause
Artery width	3	3	Positive ΔAVR	Artery constant with CO_2
Vein width	3	4		Vein narrower with CO_2

**D**	CO_2	Air	$\Delta AVR = \Delta AVR_{CO2} - \Delta AVR_{Air}$	Cause
Artery width	4	3	Positive ΔAVR	Artery wider with CO_2
Vein width	4	4		Vein constant with CO_2

Table S4) Demographic and imaging variables compared between participants with negative or positive change in:
A) Artery-vein ratio with CO_2 (ΔAVR)
B) Central retinal vein equivalent with CO_2 (ΔCRVE). Negative ΔCRVE indicates venular narrowing in response to CO_2.

**A**	**ΔAVR ≤ 0 n=17**	**ΔAVR > 0 n=27**	**P-value**
Age, years, mean (SD)	68.2 (9.3)	66.5 (8.4)	0.55
Male, %	65	81	0.28
Diabetes, %	12	11	1.00
Hypertension, %	76	78	1.00
Hypercholesterolaemia, %	65	59	0.76
Ischaemic Heart Disease, %	6	11	1.00
Smoking History (%): never	59	33	0.34
ex-greater 1yr	24	41	
ex-less 1yr	6	4	
current	12	22	
Alcohol Excess, %	18	22	1.00
Alcohol, Units per week, median (IQR)	6 (1 to 10)	14 (1 to 10)	0.39
Systolic BP, mmHg, mean (SD)	152 (15)	135 (14)	**<0.01**
Diastolic BP, mmHg, mean (SD)	81.4 (8.6)	78.5 (8.2)	0.29
Lacunar subtype, %	71	59	0.53
Days since index stroke by study scan, median (IQR)	95 (54 to 1351)	213 (56 to 1428)	**<0.01**
White matter hyperintensity volume (ml), median (IQR)	17.5 (6.4 to 33.7)	7.9 (5.1 to 14.1)	**0.05**
WMH volume (% of intracranial volume), median (IQR)	1.26 (0.45 to 2.76)	0.56 (0.32 to 1.01)	**0.04**
Total Fazekas Score (%): 0	0	0	**<0.01**
1	12	4	
2	24	52	
3	0	19	
4	18	19	
5	12	7	
6	36	0	
CRAE, pixels, median (IQR)	30.7 (28.9 to 32.6)	30.8 (28.3 to 32.7)	0.79
CRVE, pixels, median (IQR)	38.5 (37.2 to 40.8)	41.8 (38.4 to 44.5)	**0.03**
AVR, pixel ratio: CRAE/CRVE, median (IQR)	0.79 (0.75 to 0.86)	074 (0.69 to 0.77)	**0.01**
ΔCRAE, pixels, median (IQR)	-0.27 (-2.1 to 0.36)	-0.31 (-1.35 to 0.99)	0.49
ΔCRVE, pixels, median (IQR)	2.55 (1.36 to 2.92)	-2.15 (-6.85 to -0.54)	**<0.01**
ΔAVR, pixel ratio, median (IQR)	-0.05 (-0.1 to -0.04)	0.03 (0.02 to 0.09)	**<0.01**
Deep Grey Matter CVR Magnitude, %/mmHg, median (IQR), in n=47	0.11 (0.09 to 0.17)	0.15 (0.11 to 0.19)	0.14
White Matter CVR Magnitude %/mmHg, median (IQR), in n=47	0.05 (0.03 to 0.06)	0.06 (0.05 to 0.08)	0.07

P-values refer to unpaired t-test for continuous variables reported as mean (SD), and Fisher exact test for categorical variables. Mann-Whitney U test was used for most variables reported as median (IQR), except for count variables: days since index stroke and units of alcohol per week. These were analysed with Poisson regression.

B	ΔCRVE ≤ 0<br>n=24	ΔCRVE > 0<br>n=20	P-value
Age, years, mean (SD)	66.5 (7.3)	67.8 (10.3)	0.64
Male, %	79	70	0.5
Diabetes, %	8	15	0.6
Hypertension, %	83	70	0.5
Hypercholesterolaemia, %	62	60	1
Ischaemic Heart Disease, %	12	5	0.6
Smoking History (%): never	29	60	0.2
ex-greater 1yr	46	20	
ex-less 1yr	4	5	
current	21	15	
Alcohol Excess, %	8	35	0.06
Alcohol, Units per week, median (IQR)	3.5 (1 to 15.8)	10 (1 to 24.8)	**<0.01**
Systolic BP, mmHg, mean (SD)	133 (124 to 144)	146 (141 to 160)	**<0.01**
Diastolic BP, mmHg, mean (SD)	79.1 (74 to 83.2)	80.3 (76.9 to 86.1)	0.5
Lacunar subtype, %	71	55	0.4
Days since index stroke by study scan, median (IQR)	1347 (57 to 1523)	94 (54 to 480)	**<0.01**
White matter hyperintensity volume (ml), median (IQR)	8.0 (5.1 to 17.4)	13.5 (6.2 to 32.8)	0.3
WMH volume (% of intracranial volume), median (IQR)	0.59 (0.33 to 1.2)	0.89 (0.4 to 2.5)	0.3
Total Fazekas Score (%): 0	0	0	**0.05**
1	4	10	
2	50	30	
3	17	5	
4	21	15	
5	8	10	
6	0	30	
CRAE, pixels, median (IQR)	30.8 (28.6 to 32.7)	30.0 (28.4 to 32.8)	0.8
CRVE, pixels, median (IQR)	42.5 (39.2 to 44.9)	38.5 (36.8 to 40.0)	**<0.01**
AVR, pixel ratio: CRAE/CRVE, median (IQR)	0.7 (0.7 to 0.8)	0.78 (0.8 to 0.8)	**<0.01**
ΔCRAE, pixels, median (IQR)	-0.9 (-2.6 to -0.0)	0.51 (-0.4 to 1.7)	**<0.01**
ΔCRVE, pixels, median (IQR)	-2.8 (-7.0 to -1.3)	2.58 (1.5 to 3.1)	**<0.01**
ΔAVR, pixel ratio, median (IQR)	0.03 (0.01 to 0.11)	-0.04 (-0.09 to 0.00)	**<0.01**
Deep Grey Matter CVR Magnitude, %/mmHg, median (IQR), in n=47	0.16 (0.1 to 0.2)	0.12 (0.1 to 0.14)	**0.05**
White Matter CVR Magnitude %/mmHg, median (IQR), in n=47	0.06 (0.05 to 0.08)	0.04 (0.03 to 0.06)	**0.02**

P-values refer to unpaired t-test for continuous variables reported as mean (SD), and Fisher exact test for categorical variables. Mann-Whitney U test was used for most variables reported as median (IQR), except for count variables: days since index stroke and units of alcohol per week. These were analysed with Poisson regression.
